# Hepatocyte delivery of miR-34b/c reduces hepatic stellate cell activation and improves liver fibrosis

**DOI:** 10.1016/j.omtn.2025.102593

**Published:** 2025-06-09

**Authors:** Pasquale Piccolo, Rosa Ferriero, Claudia Perna, Edoardo Nusco, Marcello Monti, Rossella De Cegli, Anna Barbato, Nicolina Cristina Sorrentino, Maria Teresa Viscomi, Marica Cariello, Antonio Moschetta, Severo Campione, Nicola Brunetti-Pierri

**Affiliations:** 1 Telethon Institute of Genetics and Medicine, Pozzuoli, Italy; 2 Department of Clinical Medicine and Surgery, University of Naples Federico II, Naples, Italy; 3 Department of Life Science and Public Health, Section of Histology and Embryology, Università Cattolica del Sacro Cuore, Rome, Italy; 4 Fondazione Policlinico Universitario “A. Gemelli”, IRCCS, Rome, Italy; 5 Department of Interdisciplinary Medicine, University of Bari “Aldo Moro”, Bari, Italy; 6 Pathology Unit, “A. Cardarelli” Hospital, Naples, Italy; 7 Department of Translational Medicine, Federico II University of Naples, Naples, Italy; 8 Scuola Superiore Meridionale (SSM, School of Advanced Studies), Genomics and Experimental Medicine Program, University of Naples Federico II, Naples, Italy; 9 Institute of Endotypes in Oncology, Metabolism and Immunology “G. Salvatore”, Naples, Italy

**Keywords:** MT: Non-coding RNAs, liver fibrosis, AAV, miR-34b/c, hepatic stellate cells, microRNA, therapy

## Abstract

Liver fibrosis is a major health problem worldwide and currently available treatments are only supportive. The microRNA-34 (miR-34) family is upregulated in response to chronic liver injuries, and miR-34b/c downregulates the platelet-derived growth factor signaling receptors. Mice deleted of miR-34b/c were found to be more susceptible to liver fibrosis. Adeno-associated viral (AAV) vector-mediated hepatocyte-specific expression of miR-34b/c ameliorated liver fibrosis/cirrhosis in mice. Interestingly, expression of miR-34b/c into hepatocytes inhibited hepatic stellate cell activation*,* although no evidence of miR-34b/c expression or transfer from hepatocytes was found into hepatic stellate cells. In conclusion, these findings support delivery of miR-34b/c as anti-fibrotic treatment and may pave the way toward the development of novel microRNA (miRNA)-based therapies against hepatic fibrosis.

## Graphical abstract

**Figure:**
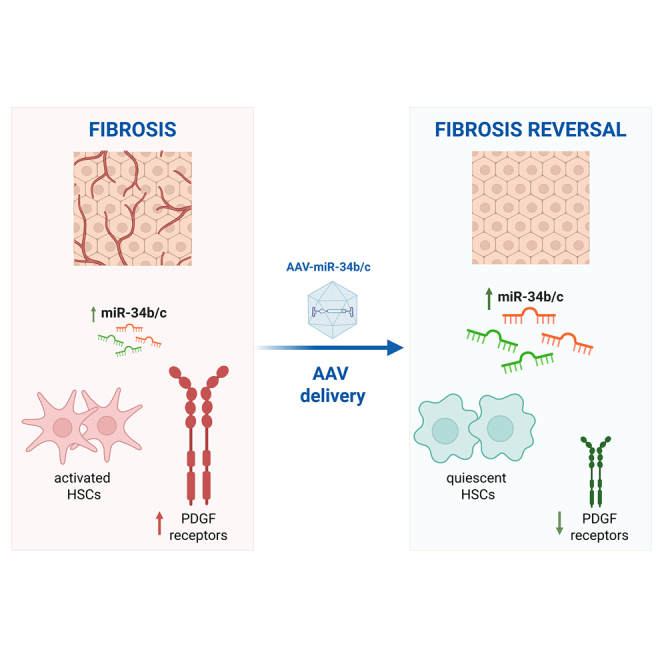

## Teaser

Brunetti-Pierri and colleagues found that mice deleted of miR-34b/c are more susceptible to liver fibrosis. Next, they showed that AAV vector-mediated hepatocyte-specific delivery of miR-34b/c ameliorated liver fibrosis/cirrhosis in mice. Therefore, delivery of miR-34b/c has potential for treatment of hepatic fibrosis.

## Introduction

Liver fibrosis is the deposition of scar tissue in the hepatic parenchyma induced by chronic injury from a variety of causes. Liver fibrosis can progress into cirrhosis that affects the organ architecture and functions with aberrant vasculature and regenerative nodules that result in portal hypertension, organ failure, and hepatocellular carcinoma. Liver cirrhosis is a leading cause of morbidity and mortality worldwide with very limited treatment options.^1^ Currently available treatments for liver fibrosis and cirrhosis are indeed only supportive and liver transplantation is the only life-saving option in severe cases.

Several microRNAs (miRNAs) have been found to regulate liver fibrosis, particularly through activation of hepatic stellate cells (HSC) and their *trans*-differentiation into myofibroblast cells, a key step in liver fibrosis. The miRNAs encapsulated within extracellular vesicles can be secreted and act as paracrine or endocrine effectors, particularly during liver damage.^2^ Few miRNAs have been found to have profibrotic roles such as the miR-21, whereas others such as miR-29a, miR-214, and miR-223 were found to have anti-fibrotic activity.^3^^,^^4^^,^^5^^,^^6^^,^^7^^,^^8^^,^^9^^,^^10^^,^^11^

We previously found that miR-34b-5p and miR-34c-5p (henceforth miR-34b and miR-34c, respectively) are upregulated in mouse models of liver fibrosis and prevent fibrosis caused by hepatic expression of mutant Z α1-antitrypsin through inhibition of platelet-derived growth factor (PDGF) signaling in a mouse model of liver disease due to alpha1-antitrypsin deficiency.^12^ Besides fibrosis, the miR-34 family has been involved in cancer and various other human diseases.^13^^,^^14^ In the present study, we show that mice deleted for the miR-34b/c develop exaggerated liver fibrosis in response to pro-fibrotic injuries and adeno-associated viral (AAV) vector-mediated delivery of miR-34b/c to hepatocytes inhibits HSC activation and has anti-fibrotic activity *in vivo*.

## Results

### Increased liver fibrosis in mice deleted for miR-34b/c

**Figure 1:**
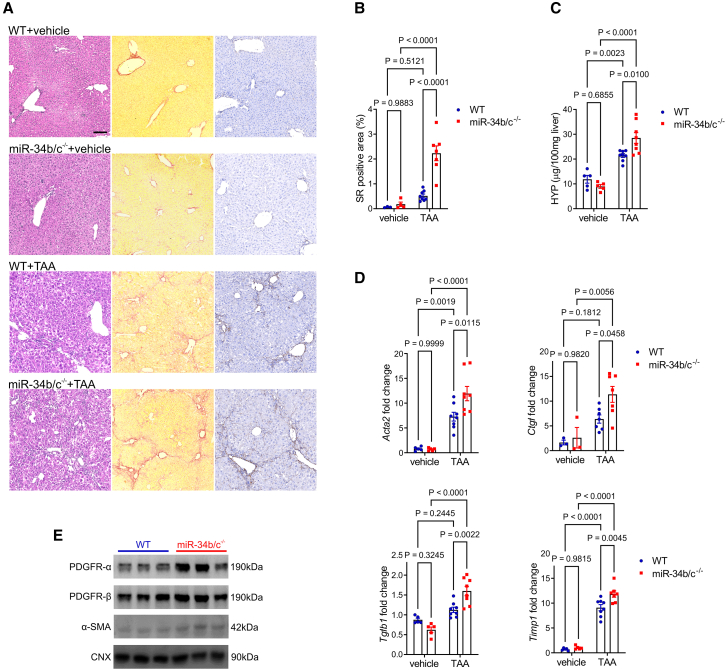
*miR-34b/c*^*−/−*^ mice are more susceptible to thioacetamide-induced liver fibrosis (A) Representative hematoxylin and eosin (left), Sirius red (SR) (central), and anti-COL1A1 (right) staining of livers from C57BL/6 wild-type (WT) and *miR-34b/c*^*−/−*^ mice treated with thioacetamide (TAA) (*n* = 8 per group) or vehicle (*n* = 5 per group). Scale bar, 100 μm. (B) Quantitative morphometry of SR staining. Data are expressed as percentage over total field area. (C) Liver hydroxyproline (HYP) content. (D) Real-time PCR of fibrosis marker genes and (E) western blot on liver extracts from WT or *miR-34b/c*^*−/−*^ mice treated with TAA (*n* = 3 per group). Calnexin (CNX) was used as loading control. Two-way ANOVA plus Tukey’s post-hoc.

**Figure 2:**
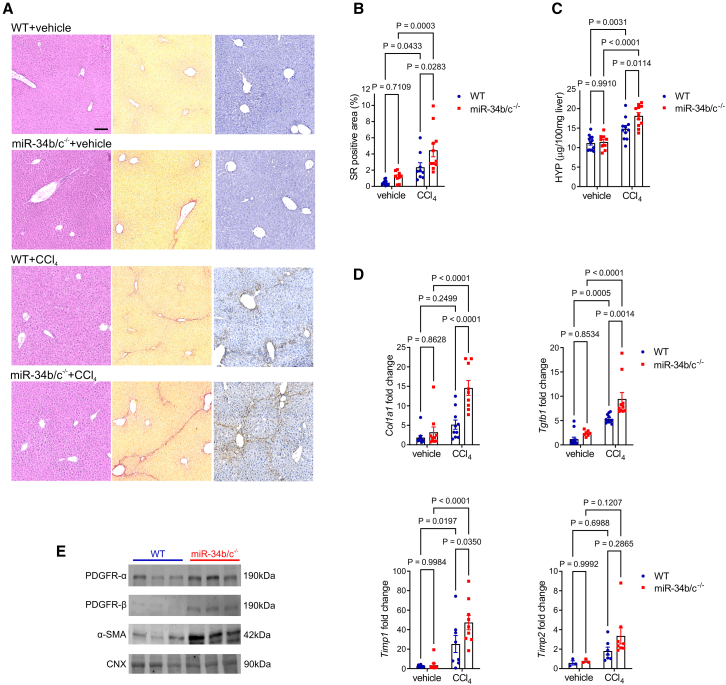
*miR-34b/c*^*−/−*^ mice are more susceptible to carbon tetrachloride-induced liver fibrosis (A) Representative hematoxylin and eosin (left), Sirius red (SR) (central), and anti-COL1A1 (right) staining of livers from C57BL/6 wild-type (WT) and *miR-34b/c*^*−/−*^ mice treated with carbon tetrachloride (CCl_4_) (*n* = 11 per group) or vehicle (*n* = 9–13 per group). Scale bar, 100 μm. (B) Quantitative morphometry of SR staining. Data are expressed percentage over total field area. (C) Liver hydroxyproline (HYP) content. (D) Real-time PCR for fibrosis marker genes, (E) Western blot analysis on liver extracts from WT and *miR-34b/c*^*−/−*^ mice treated with CCl_4_ (*n* = 4 per group). Calnexin (CNX) was used as loading control. Two-way ANOVA plus Tukey’s post-hoc.

To investigate the role of miR-34b/c in liver fibrosis, *miR-34b/c*^−/−^ and wild-type control mice were subjected to pro-fibrotic insults, including thioacetamide (TAA) and carbon tetrachloride (CCl_4_) at increasing doses for 6 and 4 weeks, respectively. Although TAA induced liver fibrosis in both *miR-34b/c*^−/−^ and wild-type control mice (Figure 1A), livers of *miR-34b/c*^−/−^ mice showed significantly increased collagen deposition, greater hydroxyproline content (Figures 1A–1C), and increased expression of fibrosis (*Acta2*, *Ctgf*, *Tgfb1*, and *Timp1*) and inflammatory marker (*Ccl2* and *Il6*) genes compared to wild-type mice (Figures 1D and S1A). Consistently, increased α and β subunits of PDGF receptor (PDGFR-α and -β) and α-smooth muscle actin (α-SMA) proteins that are markers of HSC activation were detected in TAA-treated *miR-34b/c*^−/−^ mouse livers compared to wild-type controls (Figures 1E and S1B). Compared to controls, serum alanine transaminase (ALT) activities were similarly increased by TAA in wild-type and *miR-34b/c*^−/−^ mice, whereas no differences in serum aspartate transaminase (AST) were detected among fibrotic and non-fibrotic controls (Figure S1C). Compared to controls, livers of *miR-34b/c*^−/−^ mice showed increased collagen deposition (Figures 2A and 2B), hydroxyproline content (Figure 2C), expressions of fibrosis and inflammatory genes (Figures 2D and S2A), and PDGFR-α/β and α-SMA proteins after CCl_4_ treatment (Figures 2E and S2B). Consistent with previous studies with similar dosage,^6^^,^^15^ CCl_4_ did not increase serum ALT activities compared to vehicle-treated animals (Figure S2C). Altogether, these data show that *miR-34b/c*^−/−^ mice are more susceptible to fibrotic injuries and support a protective role of miR-34b/c against liver fibrosis. Moreover, expression of miR-34c was increased in RNA from whole liver but not in the hepatocyte fraction of mice treated with TAA (Figure S3).

### Hepatic delivery of miR-34b/c reduces liver fibrosis

**Figure 3:**
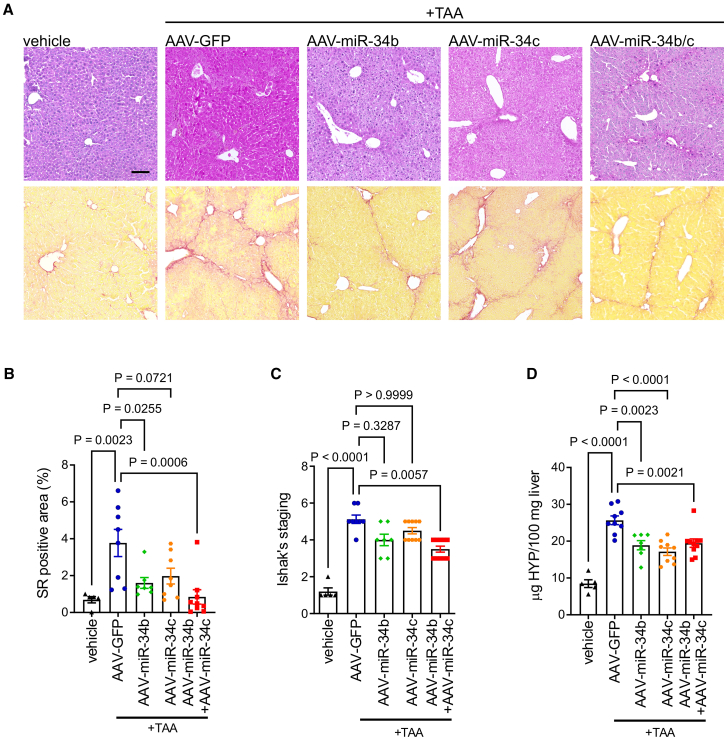
Hepatic delivery of miR-34b/c reduces TAA-induced liver fibrosis (A) Representative hematoxylin and eosin and Sirius red (SR) staining, scale bar, 100 µm; (B) quantitative morphometry of SR staining, (C) Ishak’s scores, and (D) hydroxyproline (HYP) content of livers from C57BL/6 wild-type mice treated with thioacetamide (TAA) or vehicle (*n* = 5) and injected with adeno-associated vector expressing miR-34b (AAV-miR-34b) (*n* = 7), miR-34c (AAV-miR-34c) (*n* = 9), both (AAV-miR-34b/c) (*n* = 10), or GFP as control (AAV-GFP) (*n* = 9) according to the treatment schedule shown in Figure S3A. One-way ANOVA plus Tukey’s post-hoc or Kruskal-Wallis plus Dunn’s post hoc (C only).

To investigate the potential of miR-34b/c as antifibrotic agent, we sought to deliver miR-34b/c to cells that produce it in response to injuries. To this end, mice with TAA-induced liver fibrosis were injected intravenously (i.v.) with the hepatotropic serotype 8 AAV vectors expressing the murine miR-34b or miR-34c under the control of the hepatocyte-specific thyroxine-binding globulin (TBG) promoter to potentiate the anti-fibrotic effect of miR-34c produced by hepatocytes that do not overexpress the miR-34c under TAA-induced liver fibrosis (Figure S3). Mice were treated for 12 weeks with increasing doses of TAA to induce fibrosis/cirrhosis at advanced and irreversible stages that cannot revert after TAA discontinuation.^16^ After 10 weeks of TAA, mice were injected i.v. with AAV-miR-34b, or AAV-miR-34c, or a combination of both (AAV-miR-34b/c), or an AAV expressing green fluorescent protein (GFP) as control, and sacrificed 4 weeks thereafter (Figure S4A). As expected, TAA induced extensive bridging fibrosis up to cirrhosis in livers of control AAV-GFP-injected mice (Figures 3A–3C), whereas livers of AAV-miR-34b/c-injected mice with miR-34b/c expression that was confirmed by real-time PCR (Figure S4B) showed significantly reduced fibrosis staining (Figures 3A–3C) and hydroxyproline content (Figure 3D). Moreover, expression of fibrosis marker genes in livers of mice injected with AAV-miR-34b/c was reduced compared to control AAV-GFP-injected mice and was similar to non-fibrotic livers (Figure S4C). Livers of mice injected with AAV-miR-34b showed reduction in SR positive area and hydroxyproline content and a trend in reduction of Ishak’s fibrosis score, whereas livers of mice injected with AAV-miR-34c only showed significant reduction of hydroxyproline amount compared to AAV-GFP injected mice (Figures 3A–3D). Grading of necro-inflammatory activities showed significantly increased scores in TAA-treated compared to vehicle-treated mice, but no significant reductions were detected between livers of mice injected with AAV-miR-34b, AAV-miR-34c, or combination of the two (Figure S4D). Serum ALT activities were non-significantly reduced in AAV-miR-34b/c compared to AAV-GFP-injected animals under TAA (Figure S4E).

**Figure 4:**
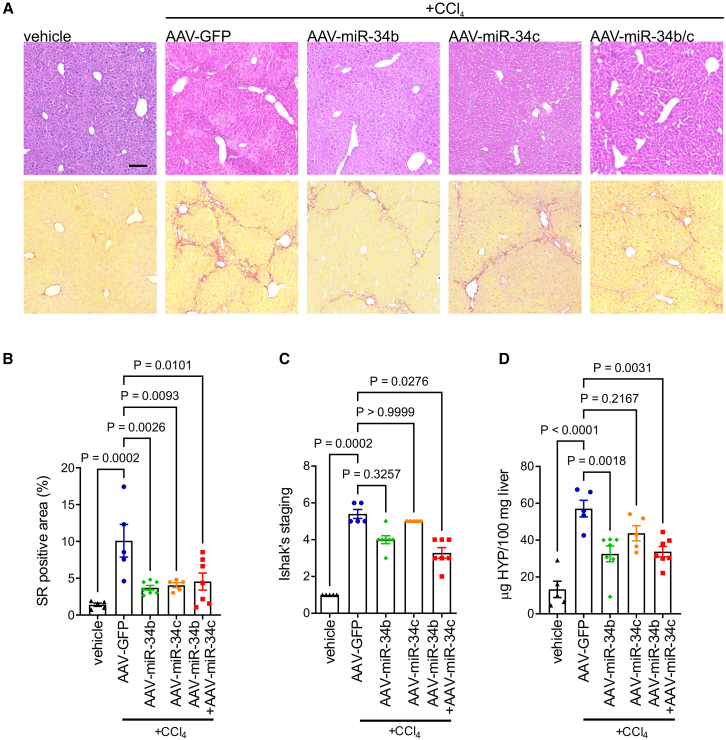
Hepatic delivery of miR-34b/c reduces CCl_4_-induced liver fibrosis (A) Representative hematoxylin and eosin and Sirius red (SR) staining, (B) quantitative morphometry of SR staining, (C) Ishak’s scores, and (D) hydroxyproline (HYP) content of livers from C57BL/6 wild-type mice treated with thioacetamide (CCl_4_) or vehicle (*n* = 5) and injected with AAV vectors expressing miR-34b (AAV-miR-34b) (*n* = 7), miR-34c (AAV-miR-34c) (*n* = 5), both (AAV-miR-34b/c) (*n* = 7), or GFP as control (AAV-GFP) (*n* = 9) according to the treatment schedule shown in Figure S4A. Scale bar, 100 μm. One-way ANOVA plus Tukey’s post-hoc or Kruskal-Wallis plus Dunn’s post hoc (C).

To confirm the anti-fibrotic effect of miR-34b/c, wild-type mice were treated for 12 weeks with increasing doses of CCl_4_.^16^ After 10 weeks of CCl_4_, mice were injected i.v. with AAV-miR-34b, or AAV-miR-34c, or AAV-miR-34b/c, or AAV-GFP, and sacrificed 4 weeks later (Figure S5A). Hepatic overexpression of miR-34b/c was confirmed by real-time PCR (Figure S5B). CCl_4_ induced advanced liver fibrosis or cirrhosis in AAV-GFP injected mice, whereas livers of mice injected with AAV-miR-34b/c showed reduced fibrosis compared to AAV-GFP injected controls (Figures 4A–4D and S5C). AAV-miR-34b and, to a lesser extent, AAV-miR-34c also ameliorated CCl_4_-induced liver fibrosis (Figures 4A–4D). Although the difference was not significant, necro-inflammatory scores were also reduced by AAV-miR-34b and AAV-miR-34b/c (Figure S5D). Serum ALT and AST activities were mildly increased or unchanged in CCl_4_-compared to vehicle-treated animals (Figure S5E), likely because CCl_4_ treatment was withdrawn for two weeks at the time of blood collection and ALT measurements (Figure S5A).

### Hepatic delivery of miR-34b/c does not ameliorate biliary fibrosis

To investigate miR-34b/c overexpression in biliary fibrosis, *Abcb4*^−/−^ mice were injected with AAV-miR-34b/c or AAV-GFP as control, and sacrificed four weeks later. *Abcb4*^−/−^ mice are a widely used model of biliary fibrosis because they recapitulate several features of human cholestatic liver diseases^17^^,^^18^^,^^19^ with moderate fibrosis by three weeks of age and advanced portal fibrosis by 10–12 weeks of age. AAV-mediated hepatic overexpression of miRNA was confirmed by qPCR (Figure S6A). Sirius red staining and hydroxyproline quantification showed no reduction of liver fibrosis in AAV-miR-34b/c compared to AAV-GFP injected animals (Figures S6B and S6C). Moreover, miR-34b/c overexpressing mice showed similar serum transaminase activities compared to controls (Figure S6D). These findings suggest that in contrast to liver fibrosis induced by TAA or CCl_4_, hepatic delivery of miR-34b/c is not effective in ameliorating biliary fibrosis of *Abcb4*^−/−^ mice. While liver fibrosis induced by TAA or CCl_4_ is mainly pericentral, fibrosis in *Abcb4*^−/−^ mice is restricted to periportal areas. Because AAV8 transduction in mice occurs mostly in hepatocytes near central veins,^20^ we next tried to deliver the miR-34b/c by the AAV-KP1 that transduces periportal hepatocytes in wild-type^21^ and *Abcb4*^−/−^ mice (Figure S7). However, the AAV-KP1 delivering miR-34b/c was also not effective at reducing liver fibrosis, despite the effective miR-34b/c overexpression (Figure S8). Taken together, these findings indicate that hepatocyte-restricted miR-34b/c overexpression either in pericentral or periportal hepatocytes did not ameliorate biliary fibrosis in *Abcb4*^−/−^ mice. Interestingly, *Pdgfra* and *Pdgfrb* expressions were very low to undetectable in the hepatocyte fraction of *Abcb4*^−/−^ mice, which failed to response to the anti-fibrotic effect of miR-34b/c delivery (Figure S9A) and enrichment of cell fractions was validated by cell specific markers (Figure S9B). In contrast, hepatocytes after CCl_4_ administration express PDGFR-α,^22^ and *Pdgfra* knock-out hepatocytes are more resistant to TAA-induced liver fibrosis,^23^ suggesting that anti-fibrotic effect of miR-34b/c requires hepatocytes expressing PDGFR-α.

### Hepatocytes overexpressing miR-34b/c reduce HSC activation

**Figure 5:**
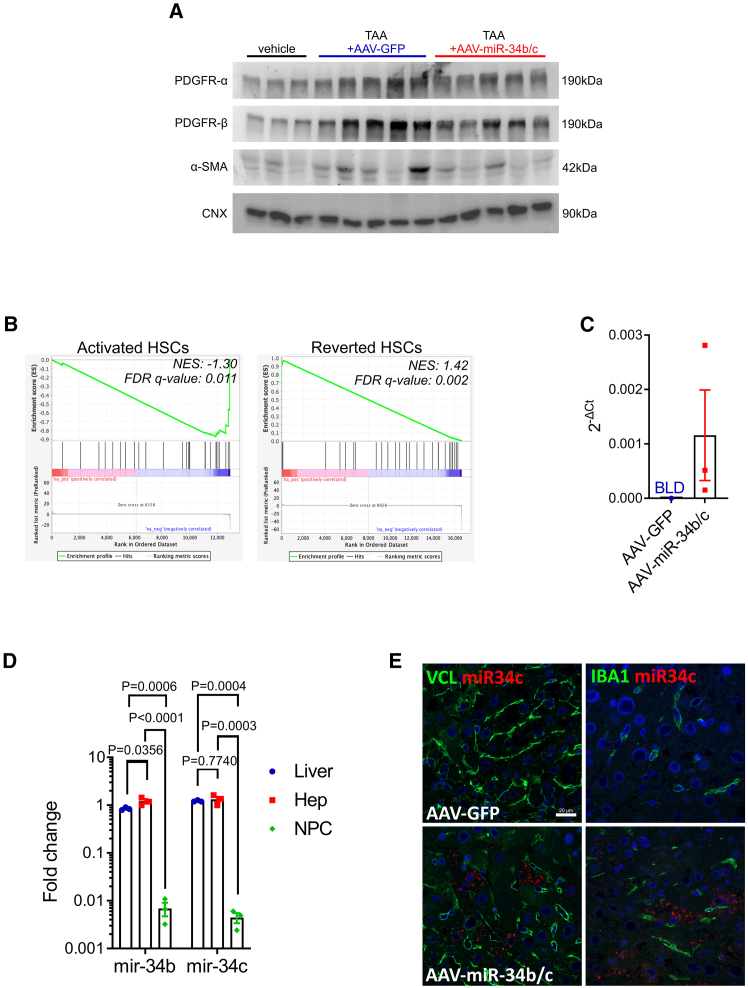
Hepatocyte-restricted miR-34b/c expression reduced hepatic stellate cell activation (A) Western blot analysis for PDGFR-α, PDGFR-β, and α-SMA on liver lysates. Calnexin (CNX) was used as loading control. (B) Enrichment plot from gene set enrichment analysis using activated and reverted HSC gene signature on transcriptomic data from livers of TAA-treated mice injected with AAV-miR-34b/c versus TAA-treated AAV-GFP-injected animals. NES, normalized enrichment score; FDR, false discovery rate. (C) miR-34c serum levels by real-time PCR (*n* = 3 per group). BLD, below the limit of detection*.* (D) miR-34b/c analysis by real-time PCR in whole livers and hepatocyte (Hep) and non-parenchymal cell (NPC) fractions from TAA-treated mice injected with AAV-miR-34b/c. One-way ANOVA plus Tukey’s post-hoc. (E) Representative images from miRNA-scope and immunofluorescence analysis on liver sections from TAA-treated mice injected with AAV-GFP or AAV-miR-34b/c using a miR-34c probe (*red*) combined with anti-vinculin (VCL) or anti-ionized calcium-binding adapter molecule 1 (IBA1) (*green*) as markers of HSC and Kupffer cells, respectively (*n* = 3 per group). Scale bars, 20 μm.

Overexpression of miR-34b/c reduced hepatic PDGFR-α/β protein and α-SMA (Figures 5A and S10) in TAA and CCl_4_ mouse models. Moreover, compared to GFP controls, transcriptomic analysis of miR-34b/c overexpressing livers followed by gene set enrichment analysis (GSEA) revealed among the downregulated genes an enrichment of genes involved in HSC activation and an enrichment of genes associated with fibrosis reversal in HSC among upregulated genes (Figure 5B). These results suggest that hepatic miR-34b/c overexpression reduces HSC activation.

Hepatocytes can actively secrete miRNAs via extracellular vesicles and this process is enhanced during liver injury.^24^ Secreted miRNAs can be taken up by adjacent or distal cells, including liver non-parenchymal cells (NPC). To investigate whether miR-34b/c overexpressed in hepatocytes from fibrotic livers is secreted, we injected TAA-treated *miR-34b/c*^−/−^ mice with AAV-miR-34b/c or AAV-GFP as control and then measured the serum miR-34c concentrations. In contrast to control mice, circulating miR-34c was detected in AAV-miR-34b/c injected mice (Figure 5C). Moreover, in the NPC fraction, the miR-34b/c was detectable at a very low level, likely as result of residual hepatocyte contamination of the cell fraction (Figures 5D and S11). In addition, miRNA-scope combined with immunofluorescence on livers failed to show any co-localization of miR-34c with either HSC (VCL)- or Kupffer cell (IBA1)-specific markers (Figure 5E). Taken together, these findings suggest that miR-34b/c expressed in hepatocytes is not taken up by other cell types in the liver and thus, it is unlikely to have paracrine effects. Serum AST and ALT activities showed no significant changes in mice injected with AAV-miR-34b/c or AAV-GFP control vector before liver injury induced by TAA or CCl_4_ for 2 and 4 weeks (Figure S12), thus suggesting that amelioration of fibrosis is not dependent on reduced hepatocellular damage.

## Discussion

In the present study, we found that mice deleted for *miR-34b/c* are more susceptible to fibrotic insults and AAV-mediated hepatic delivery of miR-34b/c has anti-fibrotic activity. The miR-34 family is composed of three members, miR-34a, -34b, and -34c, with the miR-34b and miR-34c linked as a bi-cistronic transcriptional unit.^25^ Increased expression of miR-34 family has been detected in animal models and patients with liver fibrosis.^26^^,^^27^^,^^28^^,^^29^ However, the role of miR-34 in liver fibrosis has been elusive with some evidence supporting a pro-fibrotic role^30^^,^^31^^,^^32^ while others suggesting anti-fibrotic activity.^30^^,^^33^^,^^34^ This conflicting evidence came from *in vitro* studies, often limited by the lack of co-culturing of hepatocytes with other liver cells. The present study addresses this discrepancy by showing *in vivo* anti-fibrotic activity of miR-34b/c. First, we showed that mice lacking miR-34b/c are more susceptible to liver fibrosis and delivery of miR-34b/c to hepatocytes exerts anti-fibrotic effects in two independent mouse models of liver fibrosis. Importantly, the anti-fibrotic activity was also detected at advanced stages of liver fibrosis. Next, we found that the anti-fibrotic effect of miR-34b/c does not appear to be dependent on miR-34b/c secretion or paracrine effects on other liver cells and is observed under fibrotic conditions with hepatocytes over-expressing PDGFR-α.

Liver fibrosis is the result of a maladaptive response to a vast array of hepatocellular injuries that induce integrated signaling networks, ultimately converging in activation and transdifferentiation of HSC. HSC are the central effector cells in liver fibrosis that orchestrate the deposition of extracellular matrix and fibrotic scars. Based on this model, antifibrotic therapies have been typically designed to target HSC. In contrast, although the exact mechanisms remain to be understood, this study paves the way toward a different therapeutic approach centered on hepatocytes as target cells. In our experiments, miR-34b/c expression was restricted to hepatocytes by the AAV8 tropism and the hepatocyte-specific TBG promoter. Leaky expression and paracrine uptake of miR-34b/c by liver NPCs was ruled out by miRNA-scope combined with immunohistochemistry and analysis of liver cell fractions. Compared to HSC, hepatocytes are more attractive as target cells gene-based therapies by both viral and non-viral vectors.^35^ In this proof-of-principle study, we used an AAV vector for delivery of the miR-34b/c. AAV vectors are showing promising results in clinical trials,^36^ and AAV delivery of miRNAs is under clinical investigation.^37^ However, delivery of miR-34b/c might also be performed by non-viral carriers that are increasingly used in clinical trials.^38^^,^^39^

Delivery of miR-34-b/c to hepatocytes was not effective in all three murine models of liver fibrosis investigated in this study. We found that AAV-mediated delivery of miR-34b/c exerted anti-fibrotic activity in livers with increased PDGFRα/β expression (TAA and CCl_4_ mouse models) but not in a mouse model with reduced PDGFRα/β expression (*Abcb4*^−/−^ mice). Interestingly, hepatocyte-specific PDGFR-α knockout mice are protected against liver fibrosis.^23^ Therefore, we speculate that miR-34b/c exerts its anti-fibrotic activity by inhibiting the pro-fibrotic pathway activated by PDGFRα/β in hepatocytes. We speculate that miR-34-b/c might inhibit the release of hepatocyte-derived factors that activate HSC and promote PDGFR-α/β-induced fibrosis. However, the exact mechanism underlying the anti-fibrotic effect of miR-34-b/c through PDGFRα/β in hepatocytes remains to be elucidated.

By reducing hepatocyte damage and death, hepatoprotective agents can ameliorate liver fibrosis. However, the lack of changes in serum AST and ALT activities between *miR-34b/c*^*−/−*^ mice and wild-type mice or between wild-type mice injected with AAV expressing miR-34b/c or GFP and injured with TAA or CCl_4_ suggests that the anti-fibrotic activity of miR-34b/c does not depend on a cell protective effect. The effect of miR-34b/c on inflammation is difficult to dissect, because while miR-34b/c deletion upregulates inflammatory genes during fibrosis, miR-34b/c overexpression reduced necroinflammation in CCl_4_-but not in TAA-treated animals. Comprehensive analysis of miR-34b/c targets in different liver cell types, particularly in Kupffer cells, may help to clarify if additional players involved in liver damage are regulated by miR-34b/c.

Liver fibrosis and cirrhosis are major global health problems with an estimated prevalence in the population ranging from 2% to 25%.^40^ At least in its initial stages, liver fibrosis can be reversed if the underlying insult(s) is removed. Although our understanding the pathophysiology of liver fibrosis has improved and instigated a growing number of clinical interventional trials,^41^ obeticholic acid remains the only approved anti-fibrotic drug indicated for primary biliary cholangitis at this time.^42^^,^^43^ The present study unravels the potential of a new treatment for liver fibrosis based on delivery of the miR-34b/c.

## Materials and methods

### Mouse studies

All mice received humane care according to the criteria outlined in the Guide for the Care and Use of Laboratory Animals and mouse procedures were approved by the Italian Ministry of Health. Male C57BL/6 (Charles River Laboratories) mice (6–8 weeks old), *miR-34b/c*^*−/−*^ mice^44^ (Jackson Laboratory), and 10-week-old female *Abcb4*^−/−^ mice were used for this study. *Abcb4*^−/−^ mice were maintained in *BALB/c* background,^45^ and they developed a more severe liver disease compared to other strains.^46^ TAA (Sigma-Aldrich) dissolved in phosphate buffered saline (PBS) was administered by intraperitoneal injections three times a week for up to 12 weeks at most with escalating doses, starting from 50 mg/kg/day to 400 mg/kg/day, as previously described.^16^ Briefly, escalating doses of TAA dissolved in 200 μL PBS were injected intraperitoneally three times a week, starting with 50 mg/kg (1st and 2nd dose, week 1), 100 mg/kg (2nd–5th dose, week 1–2), 200 mg/kg (6th–10th dose, week 2–4), 300 mg/kg (11th–15th dose, week 4–5), and 400 mg/kg (16th dose onwards, after week 6). CCl_4_ (Sigma-Aldrich) dissolved in corn oil (Sigma-Aldrich) was administered by gavage three times a week for up to 12 weeks at most with escalating doses, starting from 0.875 mL/kg/day to 3.25 mL/kg/day, as previously described.^16^ Briefly, mice receive CCL_4_ (50/50 vol. mixed with mineral oil) three times per week starting with 0.875 mL/kg (1st dose, week 1), 1.75 mL/kg (2nd–9th dose, week 1–4), 2.5 mL/kg (10th–23rd dose, week 4–8), and 3.25 mL/kg (after week 8). At sacrifice, mice were perfused with PBS. Serum transaminase activities were measured by Scil Vitro Vet analyzer (Scil Vet). AAV vectors were injected i.v. in the retro-orbital venous plexus in a volume of 200 μL at the dose of 1 × 10^13^ genome copies/kg.

### AAV vectors

Murine *Mir34b* and *Mir34c* regions were PCR amplified from genomic DNA of C57BL/6 mice using the following primers *Mir34b*-forward 5′-ATTTGCGGCCGCTCCGAGGGTTACTTGCACTTA-3′, *Mir34b*-reverse 5′-CGCGGATCCTTGCGGG AAGAAGGACTCG-3′, *Mir34c*-forward 5′-GCGGCCGCAGTCAATATAATGACCAAATCAGCTAAG-3′, and *Mir34c*-reverse 5′-GGATCCCAGAACAGTTCCTGCTGCTG-3′. Amplified *Mir34b* and *Mir34c* were cloned in an AAV2.1 plasmid including the TBG promoter. Serotype 8 AAV vectors were produced by triple transfection in HEK293 cells and titered as previously described (InnovaVector).^47^

### Liver assays and stainings

Hepatic hydroxyproline was measured as previously described.^16^ Briefly, homogenized liver tissue was hydrolyzed in 6 N HCl at 110°C for 16 h. Hydrolysates were filtered and assayed in citrate-acetate buffer. Samples were incubated with Chloramine-T solution (Sigma-Aldrich) for 20 min at room temperature. Next, Ehrich’s reagent (Sigma-Aldrich) was added, samples were incubated at 65°C for 20 min, and absorbance was measured at 550 nm.

After perfusion, livers were fixed in 4% paraformaldehyde for 12 h stored in 70% ethanol and embedded into paraffin blocks. For hematoxylin and eosin staining, 5-μm thick sections were rehydrated, stained with Mayer’s hematoxylin (Bio-Optica) and eosin Y (Sigma-Aldrich), dehydrated, and mounted in mounting medium (Leica Biosystems). SR staining was performed on 5-μm liver sections, which were rehydrated and stained for 1 h in picrosirius red solution (0.1% Sirius red in saturated aqueous solution of picric acid). After two changes of acidified water (0.5% acetic acid in water), sections were dehydrated, cleared in xylene, and mounted on a resinous medium. Images were captured by Axio Scan.Z1 microscope (Zeiss) and analyzed by ImageJ for quantification of SR-positive areas. Five images for each mouse were analyzed. Sections were blind evaluated by an experienced pathologist (S.C.) for fibrosis staging using Ishak scoring system evaluating the fibrosis (staging) with scores ranging from 0 (no fibrosis) to 6 (cirrhosis); necro-inflammatory activity (grading) assessing piecemeal necrosis (score 0–4); confluent necrosis (score 0–6); focal necrosis, apoptosis, and focal inflammation (score 0–4); and portal inflammation (score 0–4).^48^

To detect the miRNA transcript, we performed miRNAscope (Advanced Cell Diagnostic, USA) according to the manufacturer’s protocol.^49^ Paraffin sections were dehydrated and fixed according to the protocol, then peroxidase was applied, and antigen retrieval was performed. The miR-34c probe (no. 722391) was applied on sections; then the amplification cascade was performed according to the manufacturer’s protocol and detected by using Fast Red solution. Before miRNAscope processing, sections were incubated with primary antibodies including rabbit anti-IBA-1 antibody (1:200; no. 019–19741, FujiFilm) and mouse anti-Vinculin (1:200; no. V9131, Merck) in co-detection antibody diluent (no. 323160, Advanced Cell Diagnostic, USA). After the miRNAscope protocol, sections were incubated for 1 h at room temperature with secondary antibodies including Alexa Fluor 488 donkey anti-rabbit IgG or Alexa Fluor 488 donkey anti-mouse (1:200; Molecular Probes; Eugene, OR, USA). Sections were counterstained with DAPI, mounted with GEL/MOUNT (Biomeda, Foster City, CA, USA), analyzed with ZEISS confocal microscope LSM800 and viewed by Zen blue software.

### Western blotting

For western blotting, proteins from liver tissues were extracted in RIPA buffer according to standard procedures. Primary antibodies were diluted in TBS-T/5% milk (Bio-Rad) (Table S1). Secondary antibodies were enhanced chemiluminescence (ECL) anti-rabbit horseradish peroxidase (HRP) and ECL anti-mouse HRP (GE Healthcare). Peroxidase substrate was provided by ECL Western Blotting Substrate kit (Pierce). Analysis of band intensities was performed using Quantity One 1-D Analysis Software version 4.6.7 (Bio-Rad).

### Liver cell fraction isolation

Parenchymal and nonparenchymal cells were isolated from livers of mice by a modified protocol based on Pronase/collagenase digestion.^50^ In brief, mouse livers were perfused through the inferior vena cava with EGTA solution followed by enzymatic digestion with Pronase (Sigma-Aldrich) and then collagenase type D (Roche Applied Science). Next, livers were harvested, and liver cells were disassociated by digestion with Pronase/collagenase solution and filtered through a nylon filter (Corning) to remove undigested tissues and debris. The resulting cell suspension was centrifuged at 50 × g for 3 min at 4°C. Supernatant containing nonparenchymal cells was collected. The cell pellet was resuspended three times with 30% isotonic Percoll (Sigma-Aldrich) and centrifuged at 50 × g for 10 min at 4°C to obtain hepatocytes. The NPC liver cell fraction was obtained after the centrifugation at 600 × g for 10 min at 4°C. The cell pellet was washed with Hanks’ balanced salt solution without Ca^2+^ and Mg^2+^ one time (Gibco) and centrifuged at 70 × g for 3 min at 4°C to minimize hepatocyte contamination. The final cell suspension was centrifuged at 600 × g for 10 min at 4°C. The cell pellet was resuspended in 14% of Nicodenz (Proteogenix) and centrifuged at 1,400 × g for 20 min at 4°C. The interphase was centrifuged at 600 × g to obtain the nonparenchymal cell fraction. Purity of the fractions was evaluated by real-time PCR for population-specific markers (Table S2).

### Gene expression analyses

For gene expression analyses, total RNA from cells and livers was extracted using RNeasy mini kit (QIAGEN). RNA (1 μg) were retro-transcribed using High-Capacity cDNA Reverse Transcription Kit (Applied Biosystems). The qPCR reactions were set up using SYBR Green Master Mix and were run in duplicate on a Light Cycler 480 system (Roche). Primers are shown in Table S2. Running program was as follows: pre-heating, 5 min at 95°C; 40 cycles of 15 s at 95°C, 15 s at 60°C, and 25 s at 72°C. *B2m* was used as housekeeping genes. Data were analyzed using LightCycler 480 software version 1.5 (Roche). Hepatic expression of mmu-miR-34b-5p and mmu-miR-34c-5p were analyzed as previously described.^12^

For purification of cell-free total RNA, including miRNAs from serum, miRNeasy Serum/Plasma Advanced kit (QIAGEN) was used. Total RNA (10–20 ng) was reverse transcribed using the TaqMan MicroRNA Reverse Transcription Kit and TaqMan miRNA assay. qPCR was performed using 2 μL complementary DNA (cDNA), the TaqMan MicroRNA assay, and TaqMan Universal Master Mix II no UNG (Applied Biosystems) on a Light Cycler 480 System (Roche). The running program was as follows: preheating, 10 min at 95°C; 40 cycles of 15 s at 95°C and 60 s at 60°C. miR-16 was used as housekeeping.

Total RNA was quantified using the Qubit 4.0 fluorimetric assay (Thermo Fisher Scientific). Libraries were prepared from 125 ng of total RNA using the NEGEDIA Digital mRNA-seq research grade sequencing service^51^ (Next Generation Diagnostic srl) which included library preparation, quality assessment and sequencing on a NovaSeq 6000 sequencing system using a single-end, 100 cycle strategy (Illumina Inc.). The raw data were analyzed by Next Generation Diagnostic srl proprietary NEGEDIA Digital mRNA-seq pipeline (v.2.0), which involves a cleaning step by quality filtering and trimming, alignment to the reference genome and counting by gene.^52^^,^^53^^,^^54^ The raw expression data were normalized, analyzed and visualized by Rosalind HyperScale architecture (OnRamp BioInformatics, Inc.).^55^^,^^56^^,^^57^ False discovery rate (FDR) <0.05 was considered as statistically significant. Data were deposited in GEO with the accession no. GSE206761. GSEA was performed using the GSEA software (www.broadinstitute.org/gsea)^58^ and restricting the input to the activated^59^ and reverted^60^ HSC gene signatures.

### Statistical analyses

Statistical analyses were performed using Prism 9 software (GraphPad). One- or two-way ANOVA plus Tukey’s post-hoc or Kurskal-Wallis plus Dunn’s multiple comparison were used as statistical tests. Statistical test and experimental group size used for each experiment are reported in figure legends. Data are reported as average ±standard error.

## Data and code availability

Data were deposited in GEO with the accession no. GSE206761.

## Acknowledgments

We are grateful to Chantal Housset and Leo Van Grunsven for helpful discussion and to Frank Lammert for providing *Abcb4*^−/−^ mice. We thank Cathal Wilson for manuscript editing. R.F. is recipient of “Prof. Mario Coppo” fellowship award 2018. This work has been supported by the Alpha-1 Foundation (Gordon L. Snider Award 2016 and Research Grant 2018 to P.P.; and Research Grant 2019 to N.B.-P.), the “Federico II” University of Naples (STAR Program to P.P.), PSC Partners Seeking a Cure (P.P.), the Telethon Foundation (P.P. and N.B.-P.), and the European Union—NextGenerationEU—NRRP M6C2—Investment 2.1 Enhancement and Strengthening of Biomedical Research in the NH (PNRR-MR1-2022-12376412 to N.B.-P.).

## Author contributions

P.P. and N.B.-P. designed the study and wrote the paper. P.P., R.F., C.P., E.N., M.M., R.D.C., A.B., N.C.S., M.T.V., and M.C. performed experiments and investigations. A.M. supervised experiments in *Abcb4*^−/−^ mice. S.C. evaluated liver pathology.

## Declaration of interests

The authors have a patent related to this work WO2022184650. It is entitled “Use of microRNAs in the treatment of fibrosis.”

## Supplemental information

Document S1. Figures S1–S12 and Tables S1 and S2

Document S2. Article plus supplemental information
