# Supplementary material for: Hepatocyte delivery of miR-34b/c reduces hepatic stellate cell activation and improves liver fibrosis
Source: Mol Ther Nucleic Acids. 2025 Jun 9;36(3):102593. doi: 10.1016/j.omtn.2025.102593 (PMC12240175; doi:10.1016/j.omtn.2025.102593)
Supplement: Document S1. Figures S1–S12 and Tables S1 and S2 [file mmc1.pdf]

OMTN, Volume *36*

Supplemental information

Hepatocyte delivery of miR-34b/c reduces hepatic stellate cell activation and improves liver fibrosis

Pasquale Piccolo, Rosa Ferriero, Claudia Perna, Edoardo Nusco, Marcello Monti, Rossella De Cegli, Anna Barbato, Nicolina Cristina Sorrentino, Maria Teresa Viscomi, Marica Cariello, Antonio Moschetta, Severo Campione, and Nicola Brunetti-Pierri

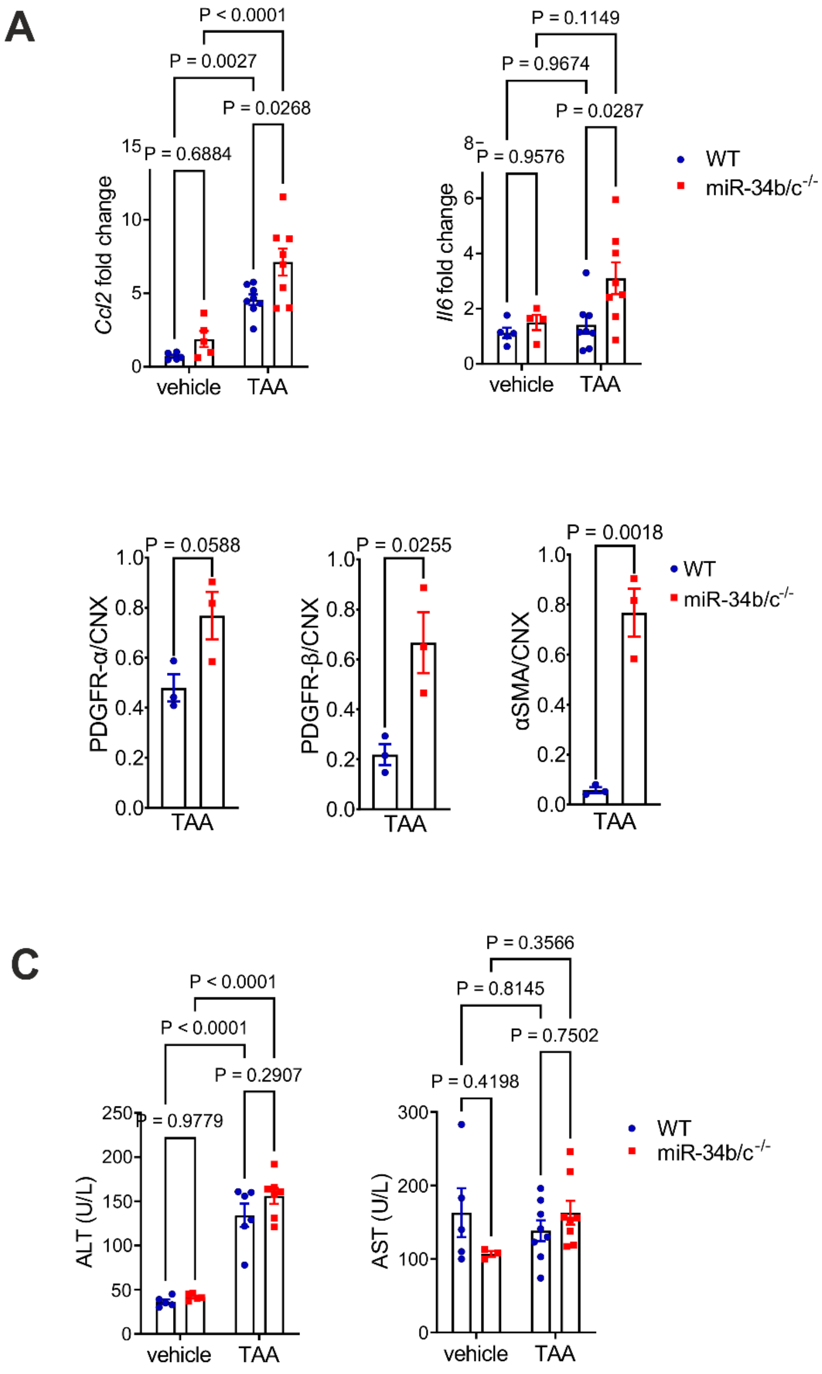

Fig. S1. Thioacetamide-induced liver fibrosis in miR-34b/c$^{-/-}$ mice. (A) Real time PCR of inflammation marker genes in livers of wild-type (WT) or miR-34b/c$^{-/-}$ mice treated with thioacetamide (TAA) (n=8 per group) or vehicle (n=5 per group). (B) Quantification of band intensities from Western blots in Fig. 1E (n=3 per group). (C) Serum alanine aminotransferase (ALT) and aspartate aminotransferase (AST) activities (n=5-8 per group). Two-way ANOVA plus Tukey's post-hoc (A, C) or *t*-test (B).

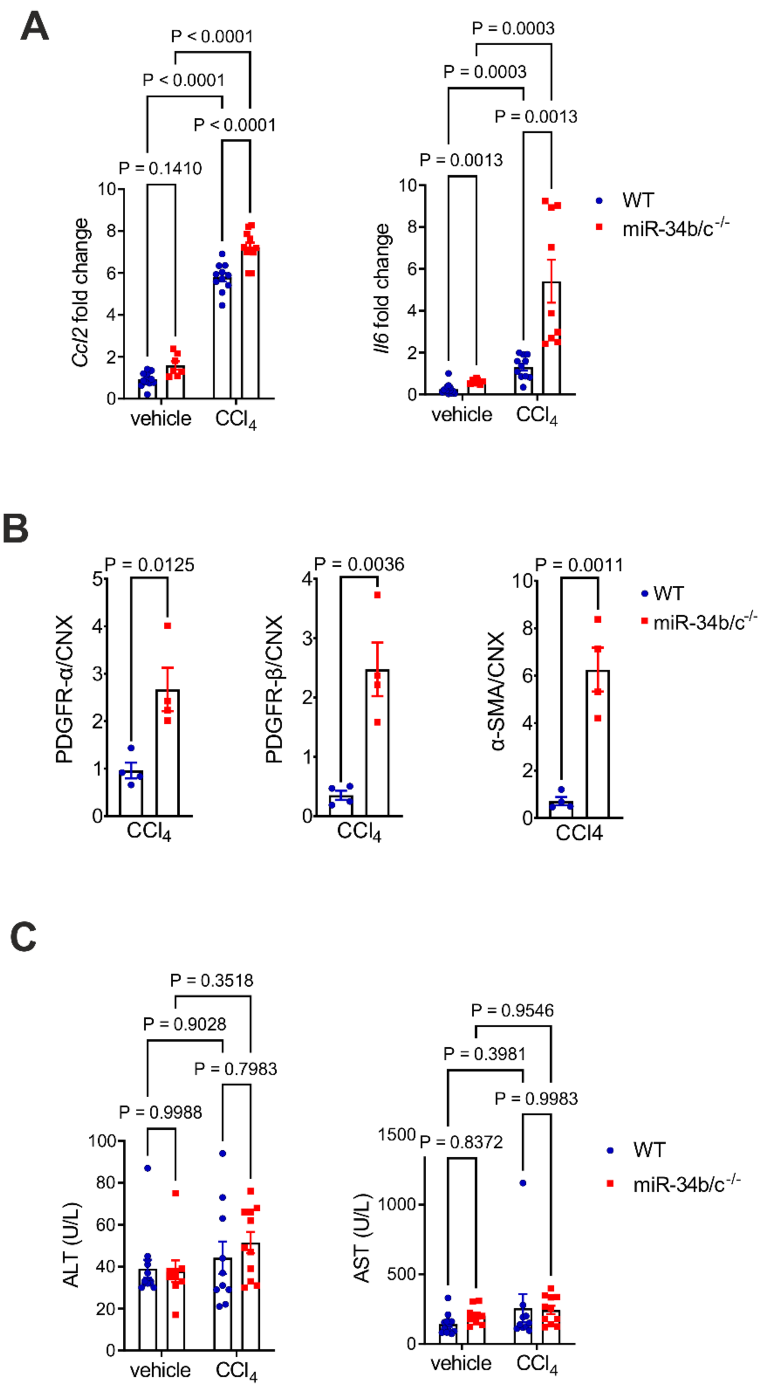

Fig. S2. Carbon tetrachloride-induced liver fibrosis in miR-34b/c$^{-/-}$ mice. (A) Real time PCR of inflammation marker genes in livers of wild-type (WT) or miR-34b/c$^{-/-}$ mice treated with Carbon tetrachloride (CCl_4) (n=11 per group) or vehicle (n=9-13 per group). (B) Quantification of band intensities from Western blots in Fig. 2E (n=4 per group). (C) Serum alanine aminotransferase (ALT) and aspartate aminotransferase (AST) activities (n=9-13 per group). Two-way ANOVA plus Tukey's post-hoc (A, C) or *t*-test (B)

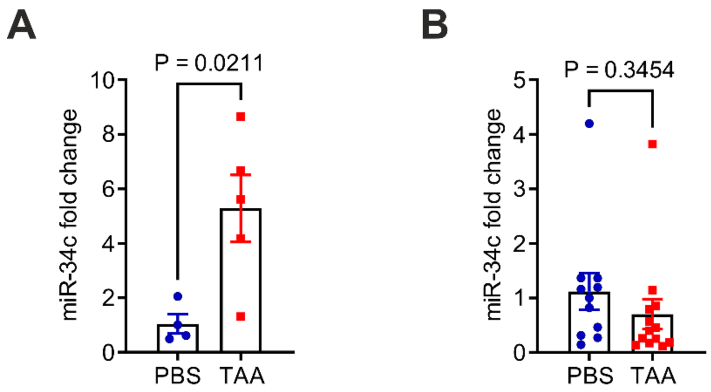

Fig. S3. Mir-34c expression in **(A)** whole liver and (**B**) hepatocyte liver fractions from wild-type mice treated with thioacetamide (TAA) or vehicle (PBS). *t*-test.

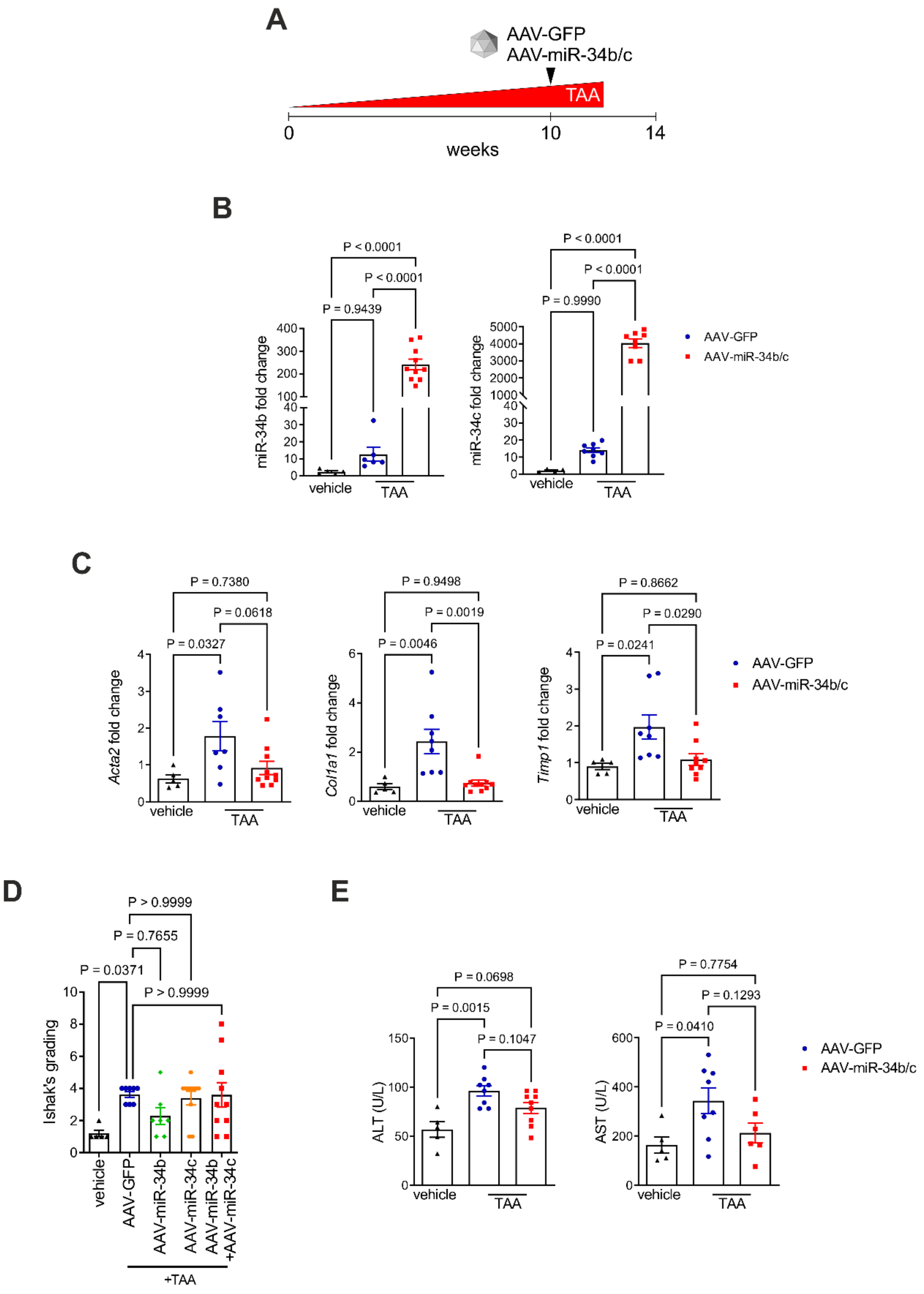

Fig. S4. Hepatic delivery of miR-34b/c ameliorates thioacetamide-induced liver fibrosis **(A)** Schematic representation of treatment schedule. **(B)** Hepatic levels of miR-34b and miR-34c by real time PCR in wild-type mice treated with thioacetamide (TAA) or vehicle (n=5) and injected with AAV vectors expressing either miR-34b/c (AAV-miR-34b/c) (n=10) or GFP as control

(AAV-GFP) (n=9). **(C)** Expression of fibrosis marker genes by real time PCR. **(D)** Necro-inflammation grading according to Ishak's scoring system. **(E)** Serum alanine aminotransferase (ALT) and aspartate aminotransferase (AST) activities. One-way ANOVA plus Tukey's post-hoc or Kurskal-Wallis plus Dunn's multiple comparison (**D** only).

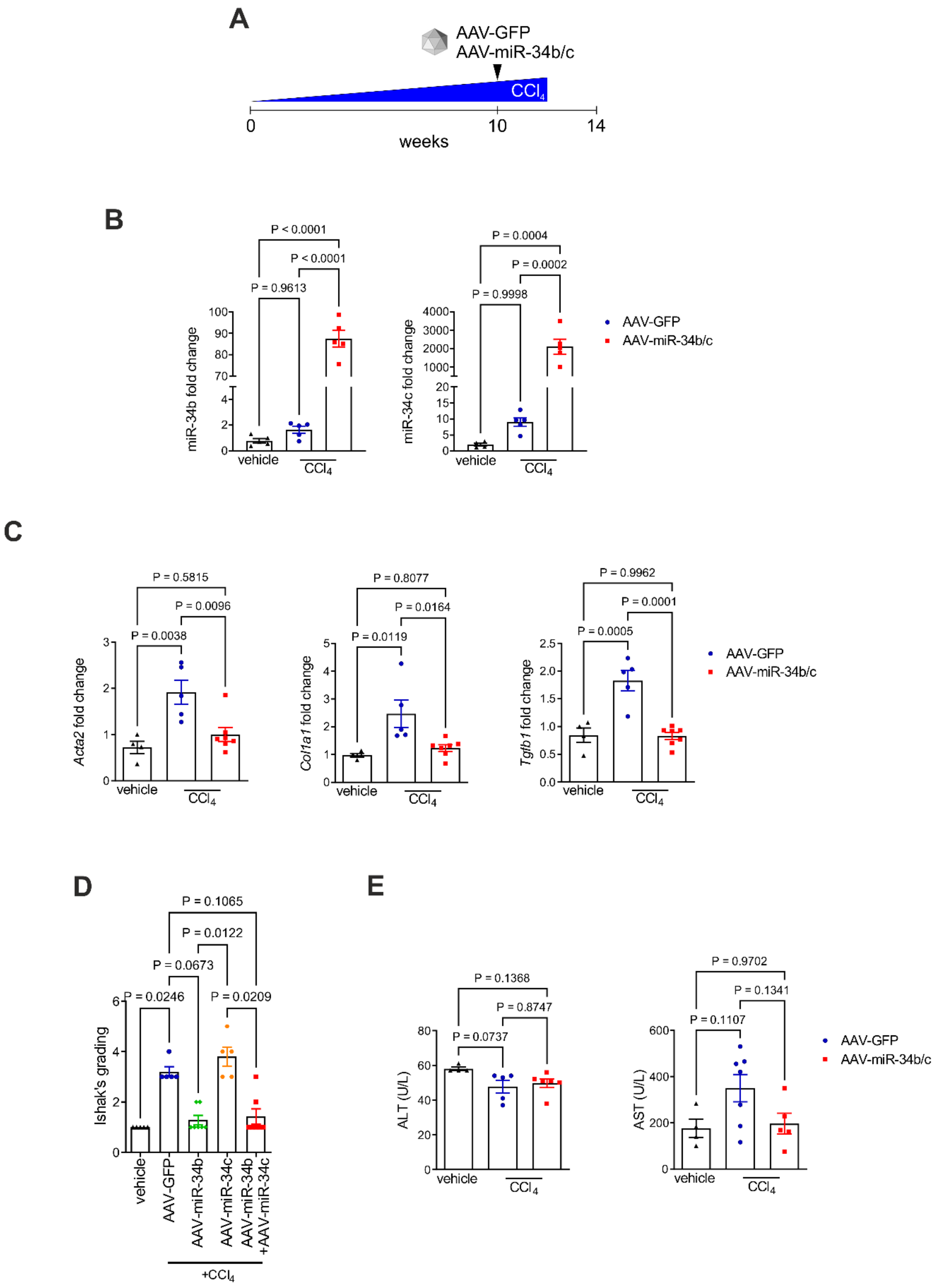

Fig. S5. Hepatic delivery of miR-34b/c ameliorates carbon tetrachloride-induced liver fibrosis. (A) Schematic representation of treatment schedule. (B) Hepatic levels of miR-34b and miR-34c

by real time PCR in wild-type mice treated with vehicle (n=5) or carbon tetrachloride (CCl_4) and injected with adeno-associated vectors expressing GFP (AAV-GFP) (n=5) or miR-34b and -34c (AAV-miR34b/c) (n= 5). (C) Expression of fibrosis marker genes by real time PCR. (D) Necro-inflammation grading according to Ishak's scoring system. (H) Serum alanine aminotransferase (ALT) and aspartate aminotransferase (AST) activities. One-way ANOVA plus Tukey's post-hoc or Kurskal-Wallis plus Dunn's multiple comparison (D only).

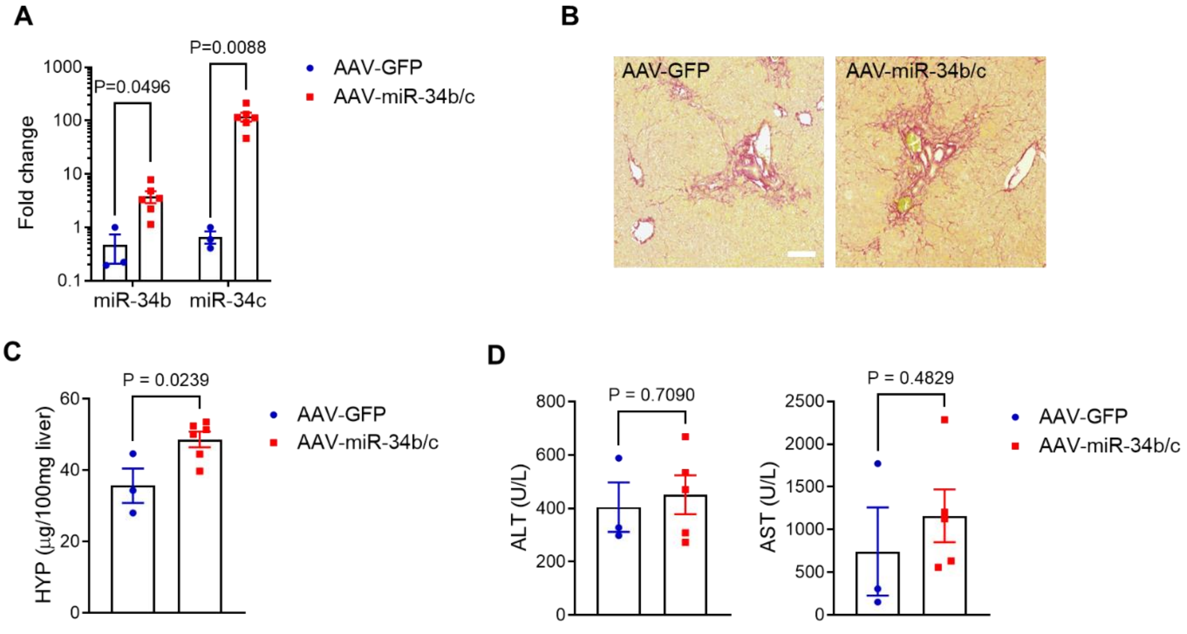

Fig. S6. AAV8-mediated overexpression of miR-34b/c does not ameliorate fibrosis in *Abcb4*$^{-/-}$ mice. 10-week-old *Abcb4*$^{-/-}$ mice were injected with AAV-miR-34b/c and AAV-GFP at the dose of $5x10^{13}$ gc/kg. Livers were collected at 4 weeks post-injection **A)** qPCR for miR-34b and miR-34c on livers from *Abcb4*$^{-/-}$ mice treated with AAV-miR-34b+AAV-miR34c or with AAV-GFP as control (n=3-5 per group) **B)** Liver sirius red staining. **C)** Hepatic hydroxyproline (HYP) content. **D)** Serum alanine aminotransferase (ALT) and aspartate aminotransferase (AST) activities. *t*-test.

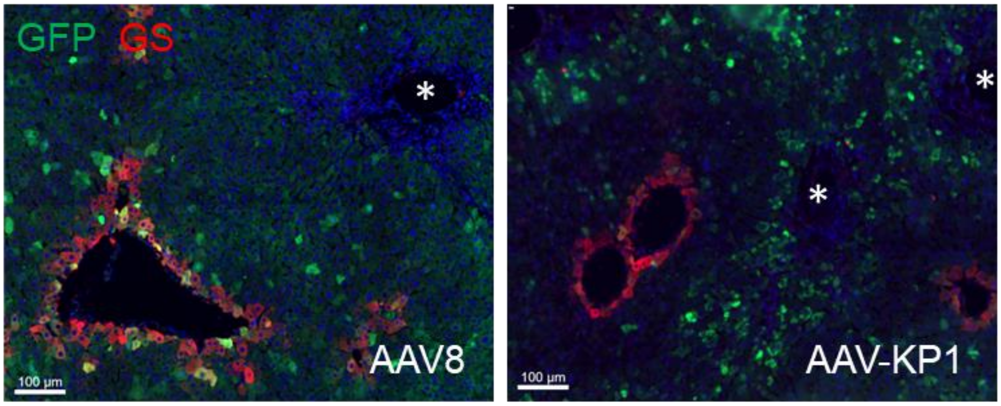

Fig. S7. Inverted zonation of liver transduction by AAV8 and AAV-KP1 vectors. 10-week-old *Abcb4*$^{-/-}$ mice were injected with AAV8-TBG-GFP and AAV-KP1-TBG-GFP vectors at the dose of $5x10^{13}$ gc/kg. Livers were collected at 4 weeks post-injection and immunostained with anti-GFP (*green*) and anti-glutamine synthetase (GS, *red*) antibodies, as a marker of pericentral hepatocytes. Nuclei were counterstained with DAPI. Portal areas are indicated by asterisks.

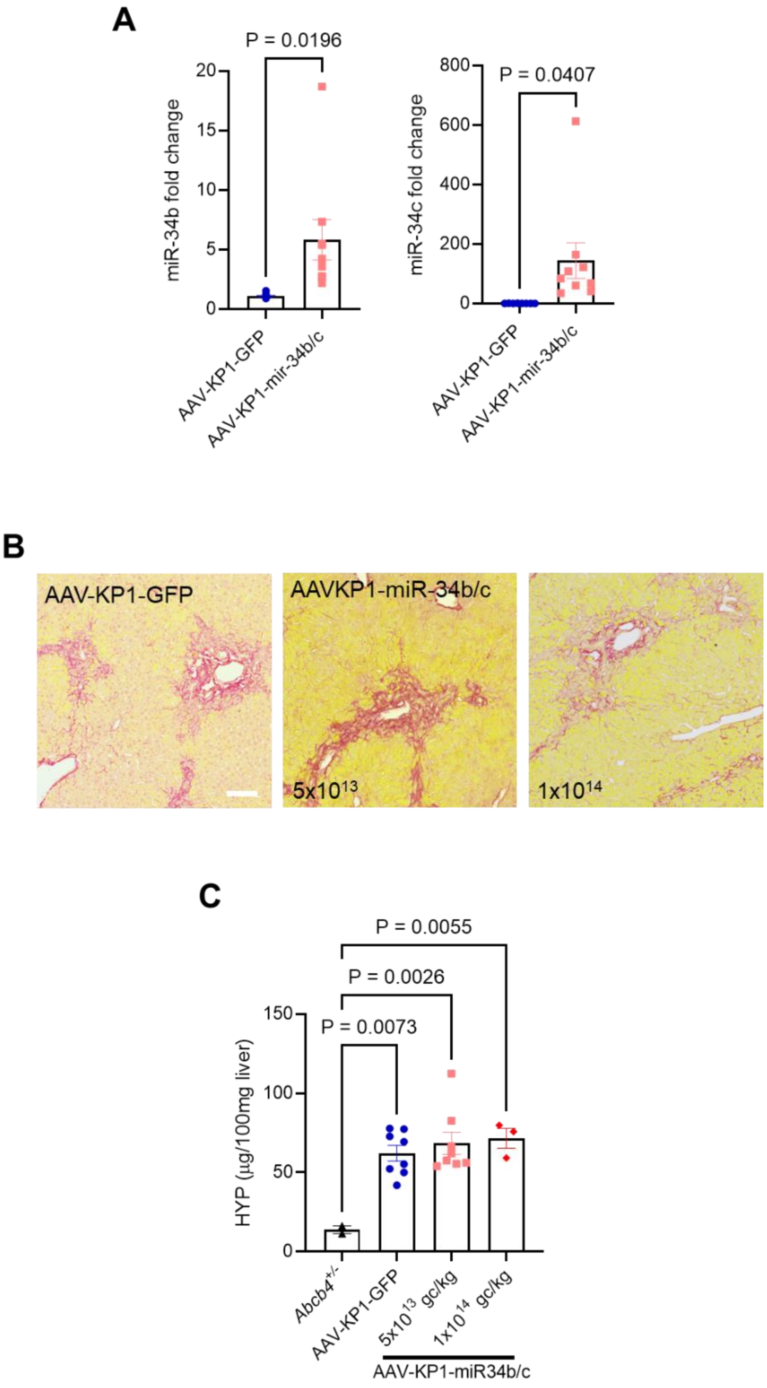

Fig. S8. AAV-KP1-delivered miR-34b/c did not ameliorate liver fibrosis in *Abcb4*$^{-/-}$ mice. 10-week-old *Abcb4*$^{-/-}$ mice were injected with AAV-KP1-miR-34b/c at the doses of 5x10^{13} and 1x10x^{14} gc/kg and with AAV-KP1-GFP as control. Livers were collected at 4 weeks post-injection **(A)** miR-34b and miR-34c expression by qPCR **(B)** Sirius red staining **(C)** Hepatic hydroxyproline (HYP) content. *t*-test (**A**) or one-way ANOVA plus Tukey's post-hoc (**C**).

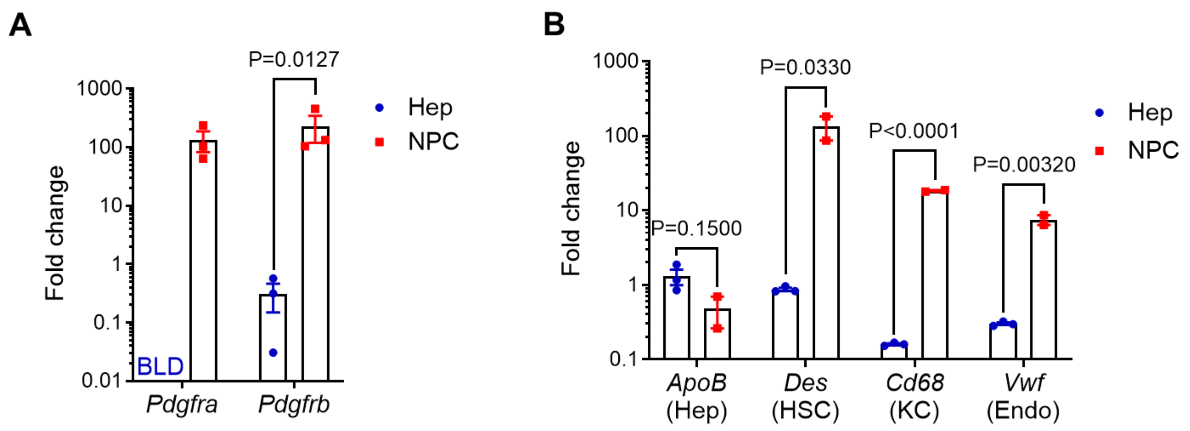

Fig. S9. *Pdgfra/b* expression in *Abcb4*$^{-/-}$ liver cell fractions. **(A)** Real time PCR analysis of *Pdgfra* and *Pdgfrb* expression in hepatocyte (Hep) and non-parenchymal cell (NPC) fractions. **(B)** Real time PCR analysis of *ApoB*, *Des*, *Cd68* and *Vwf* in hepatocyte (Hep), hepatic stellate cell (HSC), Kupffer cell (KC), and endothelial cell (Endo), respectively. *t*-test.

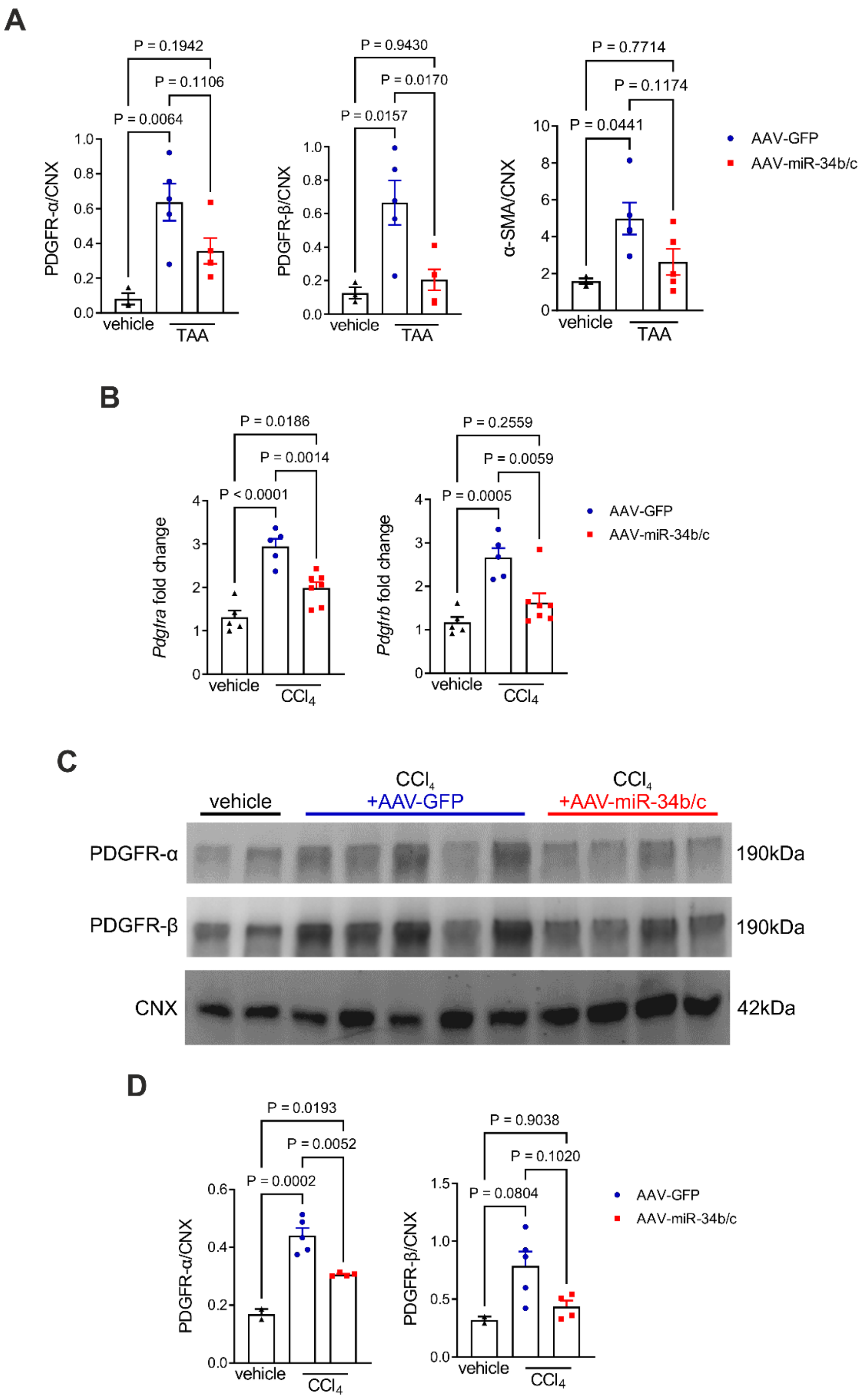

Fig. S10. Hepatic delivery of miR-34b/c reduced PDGFR-α/β expression. **(A)** Quantification of band intensities from Western blots in **Fig. 4A**. **(B)** Real time PCR analysis for *Pdgfra* and *Pdgfrb*,

(C) western blot analysis and **(D)** quantification of band intensities for PDGFR-α and PDGFR-β in whole liver lysates from C57BL/6 wild-type mice treated with vehicle (n=4) or CCl-4 and injected with adeno-associated vector expressing either miR-34b/c (AAV-miR-34b/c) (n=7) or GFP as control (AAV-GFP) (n=5). Calnexin (CNX) was used as loading control. One-way ANOVA plus Tukey's post-hoc.

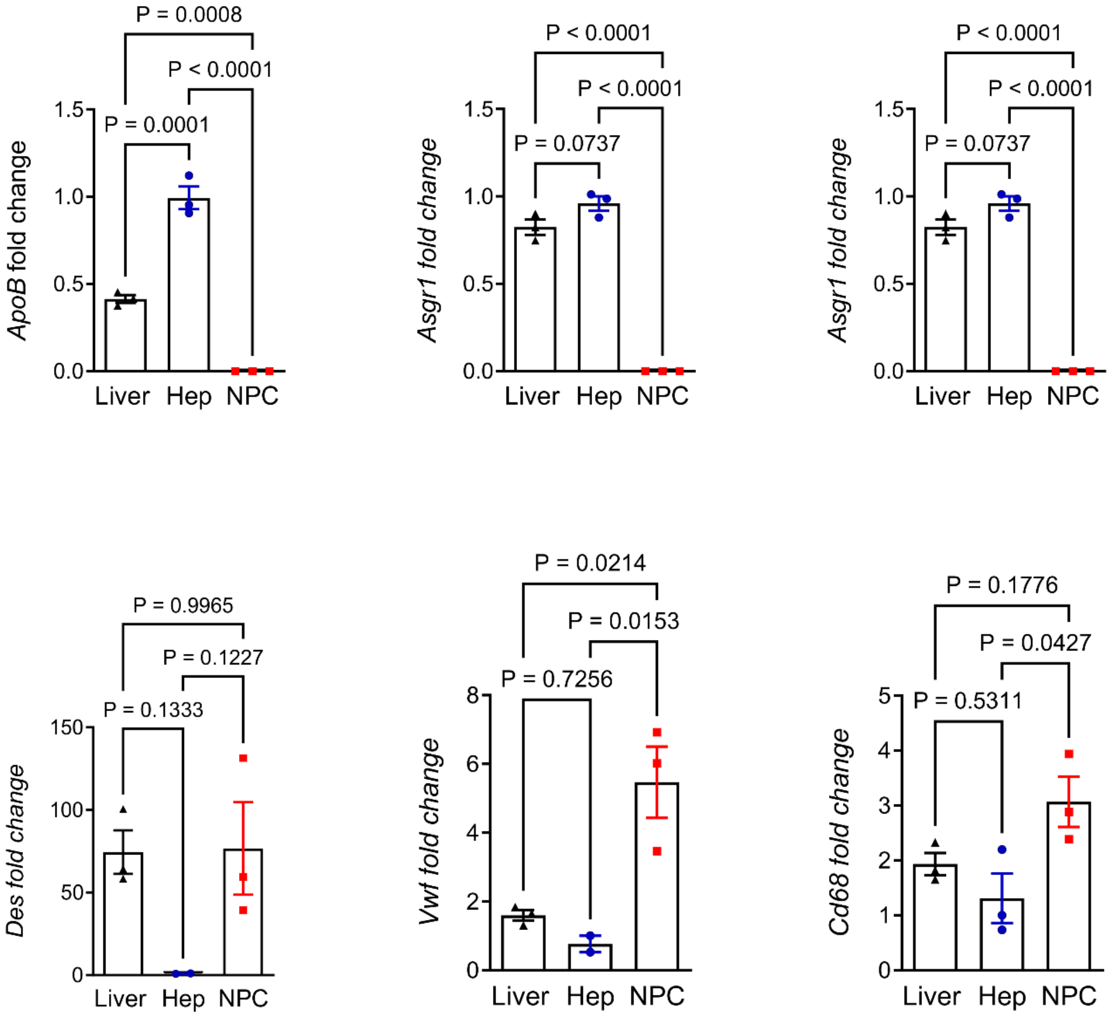

Fig. S11. Analysis of hepatocyte (*ApoB*, *Asgr1*, and *Alb*), hepatic stellate cell (*Des*), endothelial cell (*Vwf*), and Kupffer cell (*Cd68*), and markers in whole livers and hepatocyte and non-parenchymal cell (NPC) fractions from miR-34b/c$^{-/-}$ mice treated with TAA and injected with AAV-miR-34b/c. One-way ANOVA plus Tukey's post-hoc.

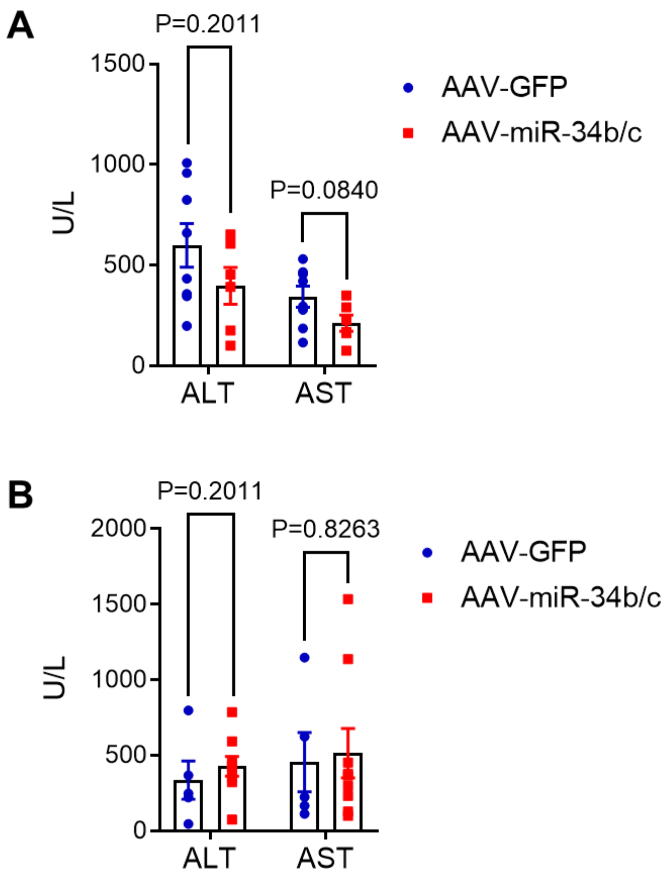

Fig. S12. Serum alanine aminotransferase (ALT) and aspartate aminotransferase (AST) activities in wild-type mice injected intravenously with AAV-miR-34b/c or AAV-GFP, and (**A)** treated 2 weeks later with increasing doses of thioacetamide and sacrificed after 2 weeks or **(B)** treated 4 weeks later with increasing doses of CCl_4 and sacrificed after 4 weeks. *t*-test.

Table S1. Primers for real time PCR.

Gene	Forward(5'-3')	Reverse(5'-3')
Acta2	CCTGGCTTCGCTGTCTACCT	TTGCGGTGGACGATGGA
Apob	AAGCACCTCCGAAAGTACGTG	CTCCAGCTCTACCTTACAGTTGA
Asgr1	TGAGCACCCAGGGAAGTAGT	CCATTGCCCCGAAATGCAG
B2m	TGGTGCTTGTCTCACTGACC	GTATGTTCGGCTTCCCATTC
Ccl2	GCTCAGCCAGATGCAGTTAA	TCTTGAGCTTGGTGACAAAAACT
CD68	CCTCGCCTAGTCCAAGGTC	GGATTCGGATTTGAATTTGGGCT
Col1a1	GCCAAGAAGACATCCCTGAA	GCCATTGTGGCAGATACAGA
Ctgf	GGGCCTCTTCTGCGATTTC	ATCCAGGCAAGTGCATTGGTA
Il6	CCGGAGAGGAGACTTCACAG	CAGAATTGCCATTGCACAAC
Pdgfra	TCCTTCTACCACCTCAGCGAG	CCGGATGGTCACTCTTTAGGAAG
Pdgfrb	AGGACAACCGTACCTTGGGTGACT	AGTTCTGACACGTACCGGGTCTC
Tgfb1	TTGCTTCAGCTCCACAGAGA	CAGAAGTTGGCATGGTAGCC
Timp1	CTCATCACGGGCCGCCTAAG	CACTGTGCACACCCCACAGC
Timp2	TCAGAGCCAAAGCAGTGAGC	GCCGTGTAGATAAACTCGATGTC

Table S2. Antibodies for Western blot analysis and immunostaining.

Antibody	Manufacturer	Dilution
anti-actin, α-Smooth Muscle (Thr183/Tyr185)	Merck (A5228)	1/1,000
anti-calnexin	Santa Cruz Biotechnology (sc-46669)	1/1,000
Anti-GFP	Abcam (ab13970)	1/800
Anti-glutamine synthetase	Abcam (ab16802)	1/200
Anti-IBA1	FujiFilm (#019-19741)	1/200
anti-PDGFR-α	Cell Signaling Technologies (#3174)	1/500
anti-PDGFR-β	Cell Signaling Technologies (#3169)	1/500
Anti-vinculin	Merck (#V9131)	1/200
